# Potential sensitivities in frequency modulation and heterodyne amplitude modulation Kelvin probe force microscopes

**DOI:** 10.1186/1556-276X-8-532

**Published:** 2013-12-18

**Authors:** Zong-Min Ma, Ji-Liang Mu, Jun Tang, Hui Xue, Huan Zhang, Chen-Yang Xue, Jun Liu, Yan-Jun Li

**Affiliations:** 1 National Key Laboratory for Electric Measurement Technology, North University of China, No. 3, Xue Yuan Road, TaiYuan, Shanxi 030051, China; 2 Key Laboratory of Instrumentation Science & Dynamic Measurement, Ministry of Education of China, North University of China, No.3, Xue Yuan Road, TaiYuan, Shanxi 030051, China; 3 Department of Applied Physics, Graduate School of Engineering, Osaka University, 2-1 Yamada-oka, Suita, Osaka 565-0871, Japan

**Keywords:** Heterodyne amplitude modulation, Frequency modulation, Kelvin probe force microscopy

## Abstract

In this paper, the potential sensitivity in Kelvin probe force microscopy (KPFM) was investigated in frequency modulation (FM) and heterodyne amplitude modulation (AM) modes. We showed theoretically that the minimum detectable contact potential difference (CPD) in FM-KPFM is higher than in heterodyne AM-KPFM. We experimentally confirmed that the signal-to-noise ratio in FM-KPFM is lower than that in heterodyne AM-KPFM, which is due to the higher minimum detectable CPD dependence in FM-KPFM. We also compared the corrugations in the local contact potential difference on the surface of Ge (001), which shows atomic resolution in heterodyne AM-KPFM. In contrast, atomic resolution cannot be obtained in FM-KPFM under the same experimental conditions. The higher potential resolution in heterodyne AM-KPFM was attributed to the lower crosstalk and higher potential sensitivity between topographic and potential measurements.

## Background

Kelvin probe force microscopy (KPFM) [1] combined with noncontact atomic force microscopy (NC-AFM) has been developed and widely used in measuring surface potential distribution and topography at atomic-scale resolution on various conductive [2,3], semiconductive [4], and insulative surfaces [5] and even on a single molecule [6]. Up to now, the origin of atomic-scale contrast in KPFM is still not fully understood, and there exists a strong controversy between several hypotheses. In the case of ionic crystals, an explanation based on short-range electrostatic forces due to the variations of the Madelung surface potential has been suggested, yet an induced polarization of the ions at the tip-surface interface due to the bias-voltage modulation applied in KPFM may be an alternative contrast mechanism [7]. In the case of semiconductors, some authors attribute atomic resolution in KPFM images to possible artifacts [8]. Some authors suggest that the local contact potential difference (LCPD) variation on a semiconductor surface is caused by the formation of a local surface dipole, due to the charge transfer between different surface atoms or charge redistribution by interaction with the AFM tip [9].

On the other hand, there are mainly three kinds of KPFM modes: frequency modulation (FM), amplitude modulation (AM) [10], and heterodyne AM-KPFM (HAM-KPFM) [11,12]. FM-KPFM, which was proposed by Kitamura et al. [13], has been shown to have the advantage of high sensitivity to short-range interactions and therefore high spatial resolution [10], and this is because the distance dependence of modulated electrostatic forces is proportional to 1/*z*^2^. AM-KPFM, proposed by Kikukawa et al. [14], has demonstrated that its advantages are its high sensitivity to potential and its ability to reduce topographic artifacts [10]; however, it also has the disadvantage of both the weak distance dependence of modulated electrostatic forces which are proportional to 1/*z*, and a serious stray capacitance effect [11,15]. As a result, the potential images we obtained using AM-KPFM are due to artifacts and not the real charge distribution. HAM-KPFM, which is given by Sugawara et al. [11] and Ma et al. [12], has been shown to almost completely remove the stray capacitance effect between the tip and the sample surface.

Consequently, to elucidate the origin of atomic resolutions of potential measurements in FM, AM, and HAM-KPFMs, it is necessary to clarify the performance of topographic and potential measurements using the three modes. Here, since the serious stray capacitance effect on LCPD images in AM-KPFM has been illustrated in the past [12], we simply discussed the potential performance in FM and HAM modes in this paper. Further, a delineation of the potential sensitivity in FM- and HAM-KPFMs, atomic-scale observations, and a comparison of the FM- and HAM-KPFMs must be further investigated experimentally.

In this study, for the first time, we investigated HAM-KPFM as a method of enabling quantitative surface potential measurements with high sensitivity by showing the contrast between FM- and HAM-KPFMs. The principle and experimental setup of FM- and HAM-KPFMs are presented. The high sensitivity of HAM-KPFM compared to FM-KPFM is experimentally demonstrated. Finally, we gave atomic resolution images of surface potential measurements on a Ge (001) surface using a W-coated cantilever in HAM-KPFM.

## Main text

### Principles of potential sensitivities in FM- and HAM-KPFMs

Firstly, we theoretically compared the performance of potential sensitivities in FM- and HAM-KPFMs. In NC-AFM, the frequency shift (∆*f*) in cantilever vibration and the energy dissipation results in an amplitude variation (∆*A*) of the cantilever's oscillation; these parameters are given by △*f* = - *f*_0_*F*_c_/(2*k*A), △*A* = *QF*_d_/*k*[16]. Here, *f*_0_, *k*, *Q*, and *A* are the resonance frequency, the spring constant, the quality factor, and the amplitude of the cantilever, respectively. *F*_c_ and *F*_d_ are the tip-sample conservative and dissipative interactions, respectively.

Therefore, the minimum detectable force for conservative interaction and for dissipative interaction are given by $\delta F_{c}=-\frac{2\mathrm{kA}}{f_{0}}\mathrm{\delta f}$ and $\delta F_{d}=\frac{k}{Q}\mathrm{\delta A}$. Here, *δf* and *δA* are the minimum detectable frequency and amplitude, respectively. For typical NC-AFM measurements in UHV, *δf* and *δA* are given by [11]: $\mathrm{\delta f}=\frac{\sqrt{12}}{\mathrm{\pi A}}f_{m}n_{\mathrm{ds}}\sqrt{B}$ and $\mathrm{\delta A}=n_{\mathrm{ds}}\sqrt{B}$, respectively. Here, *B*, *f*_m_, and *n*_ds_ are the bandwidth of the lock-in amplifier, the modulation frequency, and the deflection sensor noise of the cantilever $\left(n_{\mathrm{th}}=\sqrt{\frac{k_{B}Tf_{0}}{2\mathrm{\pi kQ}{f_{m}}^{2}}}\right)$, respectively.

Therefore, *δF*_c_ and *δF*_d_ are obtained as

(1) $$\delta F_{c}=\frac{4\sqrt{3}kf_{m}}{\pi f_{1}}n_{\mathrm{ds}}\sqrt{B},$$

(2) $$\delta F_{d}=\frac{k}{Q}n_{\mathrm{ds}}\sqrt{B}.$$

Under the typical conditions given in Table 1, *δF*_c_ is approximately 0.4pN and *δF*_d_, 0.075pN.

**Table 1:** Typical values of parameters under vacuum conditions in KPFM simulation

**Parameter**	**Unit**	**Value**
*A*	nm	5
*k*_1_	N/m	40
*k*_2_	N/m	1,600
*f*_1_	kHz	300
*f*_2_	kHz	300 × 6.3
*Q*		30,000
z_0t_	nm	6
δz_ot_	nm	0.1
*R*	nm	5
*S*	μm	38 × 225
*h*	μm	14
*f*_m_	kHz	1
*V*_ac_	V	1
*B*	Hz	200
*n*_ds_	fm/√Hz	100

In FM-KPFM, a bias voltage *V*_Bias_ = *V*_DC_ + *V*_AC_ cos *ω*_m_*t* is applied; the electrostatic force [11]$F_{\mathrm{FM}}=\frac{\pi \epsilon_{0}\mathrm{RA}}{{z_{t0}}^{2}}{V_{\mathrm{ts}}}^{2}$ at frequency *ω*_m_ is given by:

(3) $$F_{\mathrm{FM}}\approx 2\frac{\pi \epsilon_{0}\mathrm{RA}}{{z_{t0}}^{2}}\left(V_{\mathrm{CPD}}+V_{\mathrm{DC}}\right)V_{\mathrm{AC}}\cos\omega_{1}t\cos\omega_{m}t,$$

here, *V*_CPD_ is the contact potential difference (CPD) between the tip and the sample, *ε*_0_ and *R* are the dielectric constant in vacuum and the tip radius, respectively. *z*_t0_ and *A* are the average tip position and the oscillation amplitude of the cantilever, respectively.

Direct current (DC) component of the frequency shift induced by alternating current (AC) bias voltage is given by:

(4) $$\Delta f_{\text{DC}-\mathrm{FM}}=-\frac{f_{01}}{4k_{1}A}\cdot \frac{\pi \epsilon_{0}\mathrm{RA}}{{z_{t0}}^{2}}{V_{\mathrm{AC}}}^{2}.$$

From the equation $\mathrm{\Delta f}=-\frac{f_{0}}{2\mathrm{kA}}F_{c}$, the minimum detectable CPD can be described by [16]

(5) $$\delta V_{\text{CPD}-\text{FM}}=\frac{2\sqrt{6}k_{1}{z_{t0}}^{2}}{\pi^{2}\epsilon_{0}\text{RA}V_{\mathrm{AC}}}\frac{f_{m}}{f_{01}}n_{\mathrm{ds}}\sqrt{B}.$$

Note that the minimum detectable CPD in FM-KPFM is independent of the quality factor of the cantilever. Under the typical conditions in Table 1, *δV*_CPD-FM_ is approximately 15.11 mV with a *V*_AC_ of 1 V. That means that if we want to obtain a potential resolution higher than 15 mV, *V*_AC_ has to be higher than 1 V.

In HAM-KPFM, a bias voltage *V*_Bias_ = *V*_DC_ + *V*_AC_cos (*ω*_2_ - *ω*_1_) *t* is applied; the electrostatic force at frequency *ω*_2_ is given by

(6) $$F_{\text{HAM}}\approx \frac{\pi \epsilon_{0}\text{RA}}{{z_{t0}}^{2}}V_{\text{AC}}\left(V_{\mathrm{CPD}}+V_{\mathrm{DC}}\right)\cos\omega_{2}t,$$

amplitude variation induced by the electrostatic forces at frequency *ω*_2_ are described by

(7) $$\Delta A_{\text{HAM}}=\frac{Q_{2}\pi \epsilon_{0}\text{RA}}{k_{2}{z_{t0}}^{2}}\left(V_{\mathrm{CPD}}+V_{\mathrm{DC}}\right)V_{\mathrm{AC}}\cos\omega_{2}t$$

from the equation △*A* = *QF*_d_/*k*, the minimum detectable CPD is given by

(8) $$\delta V_{\text{CPD}-\text{HAM}}=\frac{\sqrt{2}k_{2}{z_{t0}}^{2}}{\pi \epsilon_{0}Q_{2}\mathrm{RA}V_{\mathrm{AC}}}n_{\mathrm{ds}}\sqrt{B}.$$

Note that minimum detectable CPD in HAM-KPFM is inversely proportional to the quality factor of the cantilever. Increasing the quality factor of the cantilever decreases the minimum detectable CPD, which means that the potential sensitivity in HAM-KPFM is enhanced. Under the typical conditions in Table 1, δ*V*_CPD-HAM_ is approximately 5.52 mV with a V_AC_ of 1 V. This value is around three times smaller than that of δ*V*_CPD-FM_. In other words, to achieve an equivalent potential resolution, the *V*_AC_ in HAM-KPFM is smaller than that in FM-KPFM.

These results show that the potential and force sensitivity detected by HAM-KPFM is higher than in FM-KPFM especially with the higher quality factor of the cantilever in vacuum condition.

### Experimental details

Next, we experimentally confirmed that the potential sensitivity of HAM-KPFM is higher than that of FM-KPFM. All experiments were performed with homemade optical interference detection UHV-AFM equipment operating at room temperature. FM-AFM was performed to provide topographic and dissipation information. The frequency shift was fed into the SPM controller (Nanonis system, SPECS Zurich GmbH, Zurich, Switzerland) as feedback to keep it constant; data acquisition and distance spectroscopy were performed by the Nanonis system.

**Figure 1:**
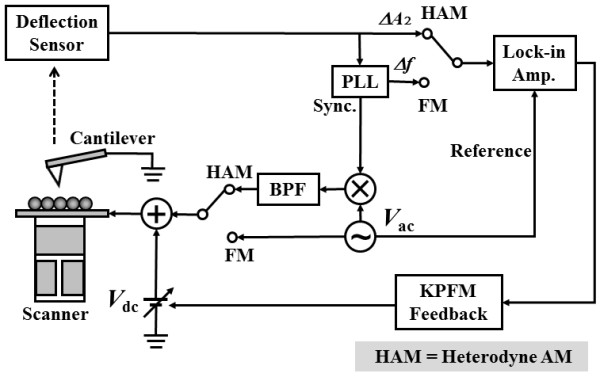
**Schematic diagram of FM- and HAM-KPFMs.** In FM-KPFM, an AC bias voltage of V_AC_cos (*ω*_m_*t*) was applied between the tip and the sample, the *ω*_m_ component of the frequency shift Δ*f*_m_ is measured with the PLL circuit and the lock-in amplifier. In HAM-KPFM, an AC bias voltage of V_AC_cos (*ω*_2_ - *ω*_1_) *t* was applied between the tip and the sample, the *ω*_2_ component of the cantilever deflection is measured with a lock-in amplifier.

Simultaneous measurements of the potential information (LCPD) were measured by FM- and HAM-KPFM, respectively. The DC bias voltage was tuned to minimize the electrostatic interaction with the bias feedback by feeding the *ω*_m_ component of the frequency shift for FM, and *ω*_2_ component of the cantilever deflection for HAM-KPFM, respectively, which was generated by the lock-in amplifier into the SPM controller. The FM- and HAM-KPFM setup diagrams are shown in Figure 1. A commercial phase-locked-loop detector (EasyPLL by Nanosurf AG, Liestal, Switzerland) was used for FM- and HAM-KPFMs. In FM-KPFM, an AC bias voltage of V_AC_cos (*ω*_m_*t*) which was generated by the commercial phase-locked-loop detector was applied between the tip and the sample, the *ω*_m_ component of the frequency shift Δ*f*_m_ is measured with the PLL circuit and the lock-in amplifier. In HAM-KPFM, an AC bias voltage of V_AC_cos (*ω*_2_ - *ω*_1_) *t* was applied between the tip and the sample, the *ω*_2_ component of the cantilever deflection is measured with a lock-in amplifier (HF2LI, Zurich Instruments, Zurich, Switzerland). The details of the experimental setup have been given in references [11,12].

A commercial silicon cantilever (Nanosensors: NCLR-W) which was detected by an optical fiber interferometer was used as the force sensor, with a spring constant *k*, resonant frequency *f*_1st_, and quality factor *Q* of about 40 N/m, 165 kHz, and 20,000, respectively, and where *f*_2nd_ is approximately 6.3 times higher than *f*_1st_ (*f*_2nd_ ≈ 1.05 MHz). The modulation frequencies in FM- and HAM-KPFM were *f*_mod-FM_ = 500 Hz, *f*_mod-HAM_ = *f*_2nd_ = 1.05 MHz. The cantilever was initially treated with an Ar^+^ ion bombardment (ion energy 700 eV, emission current: 22 μA) to remove the native oxidized layer and maintain tip sharpness. The tip was then coated by a tungsten layer with a thickness of several nanometers by sputtering the tungsten mask plate for 10 h (ion energy 2 KeV, emission current: 24 μA) to ensure sufficient tip conductivity [17]. A Ge (001) surface was chosen as the sample to determine the surface potential measurement by FM- and HAM-KPFMs. A Ge (001) specimen, cut from a Ge (001) wafer (As-doped, 0.5 to 0.6 Ω cm), was cleaned by standard sputtering/annealing cycles, that is, several cycles of Ar^+^ ion sputtering at 1 keV followed by annealing to 973 to 1,073 K.

## Discussion

### Signal-to-noise ratio measurement

**Figure 2:**
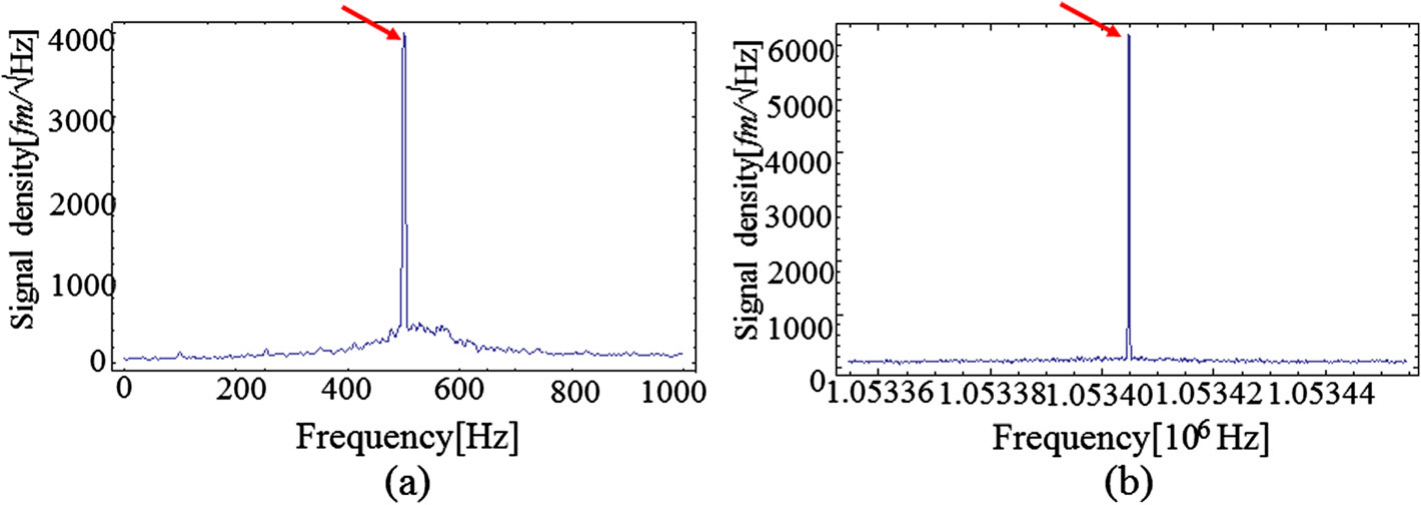
**Modulation signal spectrums of FM- and HAM-KPFM detected signals at a modulation amplitude of 150 mV (a,b).***V*_DC_ = -100 mV, A = 6.5 nm, Δ*f* = -20Hz, *f*_1st_ = 165 KHz, *f*_2nd_ =1.0089 MHz. *f*_mod_ = 500 Hz for FM-KPFM.

**Figure 3:**
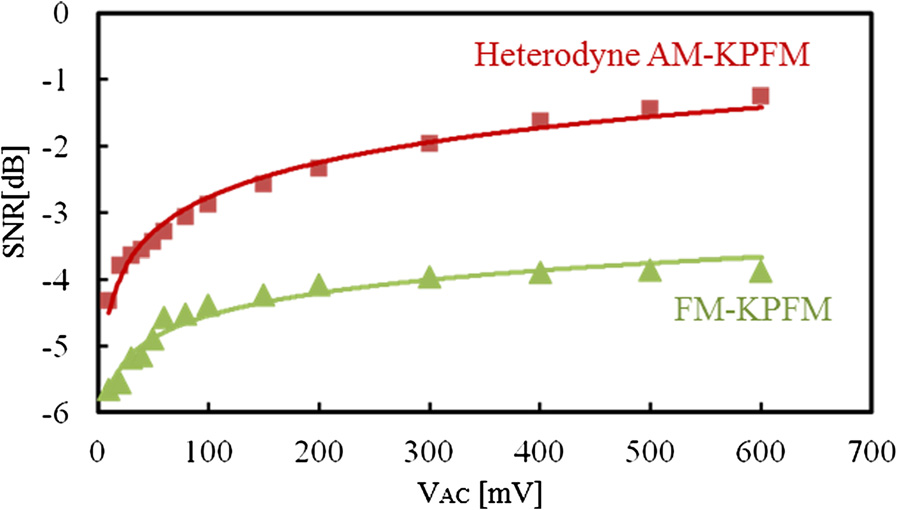
**SNRs of FM- and HAM-KPFM plotted as functions of AC bias amplitude from the density spectrums.** Given in Figure 2.

We compared the signal-to-noise ratios (SNRs) of detected signals at different bias modulation amplitudes to investigate their sensitivities to short-range electrostatic force in FM- and HAM-KPFMs. Figure 2a,b shows the noise density spectrums of the FM- and HAM-KPFMs detected signals obtained at a modulation frequency of 500 Hz for FM-KPFM and 1.05 MHz for HAM-KPFM. The bandwidth of both KPFM measurements was set to 100 Hz (narrower than that of the NC-AFM measurement). In the case of FM-KPFM (Figure 2a), signal density peak of the detected signal can reach as high as 4,000 fm/*√*Hz, while in the case of HAM-KPFM, the signal density peak of the detected signal can reach 6,000 fm/*√*Hz. These results reveal that HAM-KPFM has a higher SNR than FM-KPFM qualitatively. Figure 3 shows the *V*_AC_ amplitude as a function of the SNRs of FM- and HAM-KPFM detected signals quantitatively. SNR of FM- and HAM-KPFM detected signals monotonically increased with increasing modulation AC amplitude, and the SNR of the HAM-KPFM is higher than that of FM-KPFM with the same modulation AC amplitude. Consequently, this result shows that HAM-KPFM exhibits a higher SNR than FM-KPFM. Comparing these results with Equations (5) and (8), one can find that the minimum detectable CPD in HAM-KPFM is 1/3 that obtained in FM-KPFM in theory, in contrast, the SNR in HAM-KPFM is just 1.5 times higher than that in FM-KPFM. A possible explanation for this difference comes from the fact that quality factor of the cantilever we used was less than the simulation one. The SNR of FM-KPFM results at *V*_AC_ = 500 mV is consistent with the measurement result in literature [16].

### Surface potential measurements

We have taken potential distribution measurements on a Ge (001) surface by FM- and HAM-KPFMs using the system mentioned above. The cleaned Ge (001) surface showed a buckled dimer structure with a low, missing-dimer defect distribution. There are two main buckled dimer structures: the symmetric dimer phase p (2 × 1) configuration and the c (4 × 2) configuration [18,19]. This phase difference is caused by thermal excitation of the flip-flop motion of buckled dimers at room temperature and the interaction force between the tip apex and dimer rows [20,21]. Here, A = 6.5 nm, *V*_AC_ = 150 mV, *∆f* = -68.5Hz, and modulation frequencies in FM- and HAM-KPFMs are identical to the previous SNR measurements, respectively. The scanning area was 4 nm × 4 nm.

**Figure 4:**
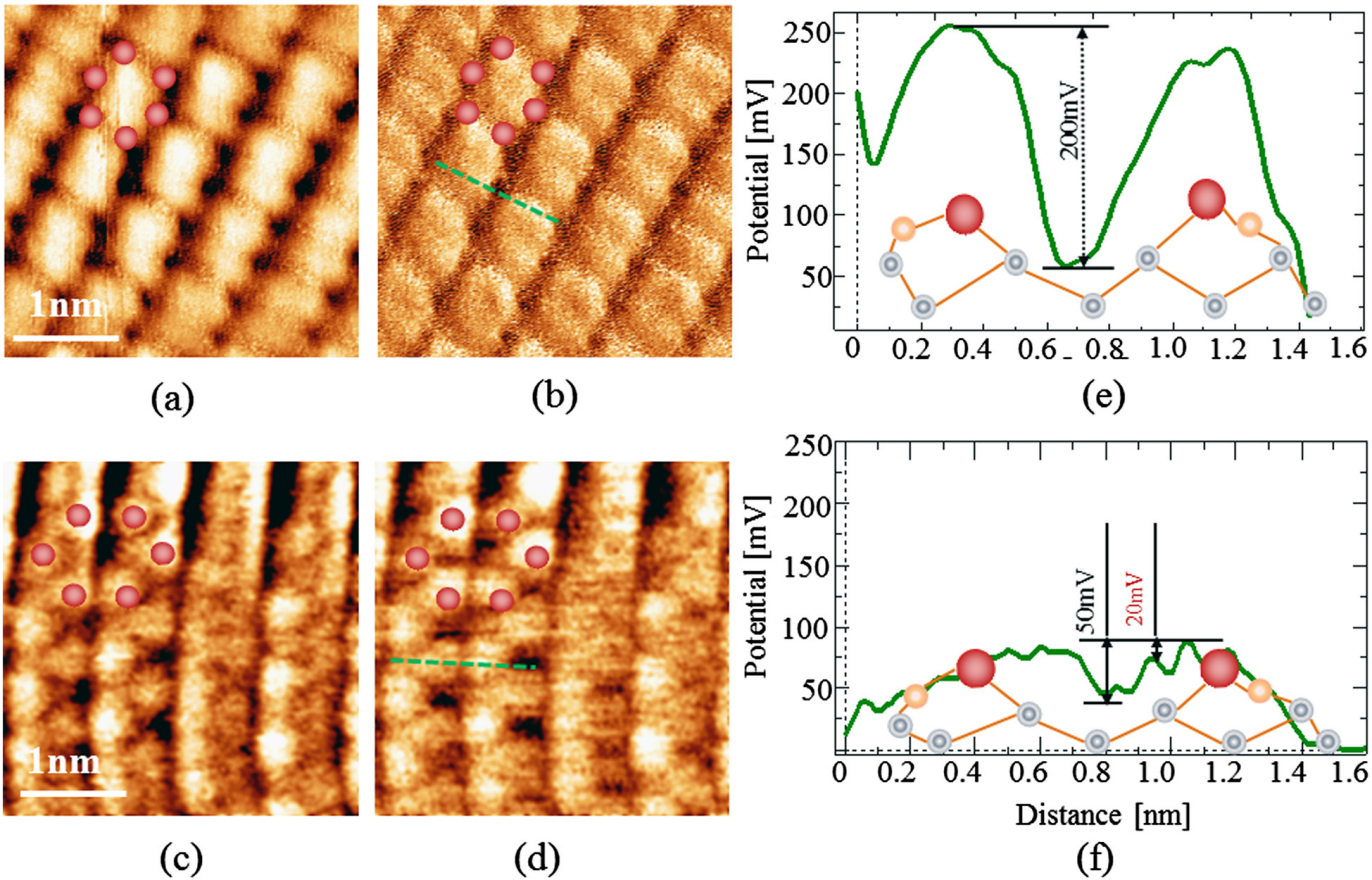
**The topographic and potential images and the potential line profiles taken by FM- and HAM-KPFMs. (a, c)** Topographic and **(b, d)** potential images taken simultaneously on the Ge (001) surface obtained by FM- and HAM-KPFMs, respectively. In the potential image, a bright (dark) spot indicates high (low) potential, which is repulsive (attractive) to electrons. **(e, f)** Cross-sectional profiles measured on the potential **(b, d)** images along the lines, respectively. The modulation frequency for FM (HAM)-KPFM is 500 Hz (1.045 MHz), respectively. Experimental parameters used in FM- and HAM-KPFMs: *A* = 6.5 nm, *V*_AC_ = 150 mV, the frequency shift was set at -6.5 Hz for AFM imaging.

Figure 4 shows the topographic and potential images and the potential line profiles taken by FM- and HAM-KPFMs. Figure 4a,c depicts topographies, and Figure 4b,d shows the corresponding potential images taken simultaneously on Ge (001) by FM- and HAM-KPFMs, respectively. From these results, it can be seen that atomic resolution cannot be observed with FM-KPFM; on the other hand, atomic resolution was obtained in HAM-KPFM in topographic and potential images. Furthermore, low frequency noise can clearly be observed in FM-KPFM while this noise disappeared in HAM-KPFM. Consequently, the potential image obtained by HAM-KPFM shows a clearer contrast than that of FM-KPFM. The reason for this is that the SNR in HAM-KPFM is higher than in FM-KPFM. This difference in potential measurements from the reference [12] between FM- and HAM-KPFM is because the steady state for FM-KPFM is usually at high voltage (*V*_DC_ approximately at 1 V) and this voltage easily makes the dimer atoms on the surface adsorbing to the tip apex to form double covalent bonding with the surface atoms. Besides, the influence of the topographic measurement seriously affects the potential images with high AC bias voltage. In contrast, for HAM-KPFM, this phenomenon can be ignored (the results are not shown here).These results demonstrated that the HAM-KPFM has a higher potential resolution and lower crosstalk than FM-KPFM.

Quantitatively, the potential line profile contrast is shown in Figure 4e,f. The minimum detectable potential in FM-KPFM was more than ten times (more than 20 mV) higher than that detected in HAM-KPFM (approximately 2 mV). The results show that HAM-KPFM can get much higher spatial resolution and potential sensitivity even with a smaller *V*_AC_ than that of FM-KPFM. The higher potential sensitivity of HAM-KPFM was explained as follows: the oscillation of the frequency shift at *ω*_1_ in FM-KPFM and the oscillation of the amplitude at *ω*_2_ in HAM-KPFM are both proportional to the gradient of the electrostatic force, whereas the quality factor in UHV for the AFM system is approximately several tens of thousands greater, and finally, that the minimum detectable electrostatic force in HAM-KPFM is smaller than in FM-KPFM according to Equations (1) and (2). Hence, the potential sensitivity in HAM-KPFM is higher than that in FM-KPFM. Further, lower crosstalk between topography and potential images in HAM-KPFM compared to that in FM-KPFM is due to the first and second resonance signals being separated from each other using low- and high-pass filters in HAM-KPFM; on the other hand, the potential and topography signals are difficult to separate because the first resonance of the cantilever was oscillated in both measurements.

In HAM-KPFM measurements, the high *V*_AC_ effect was apparently removed because small AC bias voltages were applied and the *V*_CPD_ which compensated the CPD between tip and sample is 20 to 100 mV [11,12], and this is of major importance for semiconducting samples for which voltages exceeding 100 mV may induce the band bending effect [21]. In some references, quasi-constant height mode was performed to eliminate the *V*_AC_ influence to the potential measurement [4].

## Conclusions

In summary, the potential sensitivity and crosstalk were compared in FM- and HAM-KPFM experimentally and theoretically. We demonstrated that the potential sensitivity in HAM-KPFM is higher than that in FM-KPFM theoretically. Then, we experimentally confirmed that SNRs of electrostatic force measurements, which determined the potential sensitivity in HAM-KPFM, are higher than that of FM-KPFM. Further, we applied the FM- and HAM-KPFM measurements to a Ge (001) surface under the same conditions, and atomic resolution in potential and topography images were obtained in HAM-KPFM, whereas the atomic resolution was not visible in FM-KPFM. We attribute this to the higher sensitivity and lower crosstalk in HAM-KPFM compared to the FM-KPFM. Consequently, the HAM method proposed here is a useful tool for detecting the actual potential distribution on the surface.

## Competing interests

The authors declare that they have no competing interests.

## Authors’ contributions

ZM, JM, JT, HX, and HZ carried out the calculations, performed the experiments, and drafted the manuscript with the help of CX and JL. YL participated in the design of the study and helped to draft the manuscript. All authors read and approved the final manuscript.
